# Advances in clinical application on nursing intervention for ribavirin-associated adverse events

**DOI:** 10.3389/fphar.2025.1596007

**Published:** 2025-10-28

**Authors:** Li-Li Jiang, Qing Wang, Jia-Xin Zhang, Jing-Hui Fan, Jing Li

**Affiliations:** ^1^ Department of Urology Surgery, Hongqi Hospital Affiliated to Mudanjiang Medical University, Mudanjiang, China; ^2^ Department of Pediatrics, Hongqi Hospital Affiliated to Mudanjiang Medical University, Mudanjiang, China; ^3^ Second Department of General Surgery, Hongqi Hospital Affiliated to Mudanjiang Medical University, Mudanjiang, China; ^4^ Department of Pharmacy, Hongqi Hospital Affiliated to Mudanjiang Medical University, Mudanjiang, China; ^5^ Second Department of Orthopedics and Pain, Hongqi Hospital Affiliated to Mudanjiang Medical University, Mudanjiang, China

**Keywords:** ribavirin, adverse events, nursing intervention, clinical application, advances

## Abstract

Ribavirin is a broad-spectrum antiviral drug used to treat chronic hepatitis C, respiratory syncytial virus infections, and other viral diseases. However, its clinical use is often limited by adverse events, including hemolytic anemia, gastrointestinal disturbances, and neurological symptoms, which negatively affect treatment adherence and patient well-being. Nursing interventions are essential in mitigating these adverse events through early assessment, symptom management, patient education, and multidisciplinary collaboration, ultimately improving patient outcomes. Current evidence shows that nursing interventions significantly improve treatment efficacy and quality of life. However, challenges persist, such as individual patient variability, resource constraints, and insufficient evidence-based guidelines. Future research should focus on establishing standardized nursing protocols, conducting multicenter clinical studies, exploring intelligent nursing approaches, and enhancing nursing expertise to optimize ribavirin-related adverse event management.

## 1 Introduction

### 1.1 Clinical applications of ribavirin

Ribavirin, a broad-spectrum antiviral agent, has been a key treatment for viral infections since the 1980s. It is commonly used for chronic hepatitis C virus (HCV) infection, respiratory syncytial virus (RSV) infections, and hemorrhagic fevers (Dai et al., 2021; Manothummetha et al., 2023). Previously, ribavirin combined with interferon was the standard HCV therapy before direct-acting antivirals (DAAs) were introduced (Dai et al., 2021; Zanaga et al., 2016). Despite the predominance of DAAs, ribavirin retains a well-defined role in contemporary medicine. In the realm of HCV, it is incorporated into specific DAA-based salvage regimens for patients who have experienced previous DAA treatment failure (Zuckerman et al., 2017; Wang et al., 2024). This is particularly relevant for individuals infected with HCV genotype 3, especially those presenting with baseline resistance-associated substitutions that can compromise the efficacy of certain DAA therapies (Wang et al., 2024; Poordad, 2015). Beyond patient-specific factors, ribavirin use persists in resource-limited settings where access to the full spectrum of newer DAAs remains constrained (Zuckerman et al., 2017). Furthermore, its therapeutic utility extends beyond HCV; ribavirin is employed in managing severe RSV infections in immunocompromised adults and serves as a first-line intervention for certain viral hemorrhagic fevers, including Lassa fever and Crimean-Congo hemorrhagic fever (Tejada et al., 2022; Beaucourt and Vignuzzi, 2014). Consequently, the ongoing relevance of ribavirin in these diverse clinical niches necessitates the effective management of its associated adverse events.

Adverse events (AEs) refer to unintended and harmful outcomes caused by medical interventions rather than the underlying disease, as defined by the World Health Organization and the U.S. Food and Drug Administration. AEs pose a significant threat to patient safety and healthcare quality, being closely associated with higher hospitalization rates, prolonged treatment duration, and increased healthcare expenditures (de Vries et al., 2008). Recent data indicate that up to 15% of hospitalized patients worldwide experience at least one adverse drug event, nearly 50% of which could be prevented through timely monitoring and effective intervention (Phill et al., 2014; Franceschi et al., 2008; de Lemos et al., 2021). Frontline healthcare providers, especially nurses, play a vital role in the early detection, documentation, and management of AEs, thereby enabling prompt clinical response and minimizing patient harm (Vaismoradi et al., 2020; Durham, 2015). Their responsibilities encompass active symptom surveillance, effective communication with physicians, patient education, and strict adherence to pharmacovigilance standards (Vaismoradi et al., 2020; Durham, 2015). These responsibilities become particularly crucial when managing antiviral agents such as ribavirin, which is widely associated with hematological, gastrointestinal, and neurological toxicities.

### 1.2 Prevalence and impact of ribavirin-associated adverse events (RAAEs)

Ribavirin’s clinical utility is restricted by its adverse effects. Studies indicate that more than 70% of patients experience side effects, with hemolytic anemia being the most prevalent and severe (Sato et al., 2019). Other frequent adverse events include fatigue, nausea, skin reactions, and insomnia, which reduce treatment adherence and quality of life (Sherman, 2012). Additionally, ribavirin is contraindicated in pregnancy due to its teratogenic and embryotoxic risks, complicating treatment for women of childbearing age (Sinclair et al., 2017). These adverse events increase healthcare costs, prolong hospital stays, and may lead to treatment discontinuation, negatively impacting patient outcomes.

### 1.3 Role of nursing interventions in managing drug-related adverse events

Nursing interventions play a pivotal role in reducing drug-related adverse events (Larrey et al., 2011). Research indicates that structured nursing care can significantly reduce the incidence and severity of adverse drug reactions (Jia and He, 2021; Sun, 2021). In the context of ribavirin therapy, nurses contribute to patient safety through continuous monitoring, timely dose adjustments, patient education, and psychosocial support (Liu and Wu, 2017; Zhang and Chang, 2015). Targeted interventions, including anemia management, symptom control, and personalized counseling, have proven effective in improving treatment adherence and minimizing adverse events (Liu and Wu, 2017; Zhang and Chang, 2015). These findings underscore the essential contribution of nursing to optimizing therapeutic outcomes.

In the context of RAAEs, nursing interventions refer to structured, evidence-based strategies employed to prevent, detect, and manage treatment-related toxicities. These include routine hemoglobin monitoring for early detection of hemolytic anemia, administration of folic acid, and nutritional support to mitigate hematologic toxicity (Russmann et al., 2006; Reddy et al., 2007). Dietary counseling is also implemented to address gastrointestinal symptoms such as nausea and vomiting (Russmann et al., 2006). Evidence indicates that nurse-led interventions improve treatment adherence and reduce dose adjustments. In a multicenter study, Larrey et al. demonstrated that systematic nurse-delivered education significantly enhanced treatment response rates in patients receiving peginterferon-α2a and ribavirin for chronic HCV, decreasing the necessity for dose modifications due to adverse events (Larrey et al., 2011). Similarly, Cacoub et al. reported in a real-world observational study that individualized patient education programs significantly improved adherence and reduced discontinuation rates in patients with HCV genotype 2 or 3 infections undergoing ribavirin-based therapy (Cacoub et al., 2008). These findings further highlight the multifaceted role of nursing interventions in promoting treatment safety and improving clinical outcomes.

### 1.4 Objectives and significance

Given ribavirin’s continued clinical use and its challenging adverse events profile, identifying effective nursing strategies is critically important (Liu and Wu, 2017; Zhang and Chang, 2015). This study aims to comprehensively review the current evidence on nursing interventions for RAAEs, assessing their effectiveness and clinical applicability. Additionally, it aims to identify gaps in the current research to inform future studies. This narrative review will not only improve the management of RAAEs but also offer a framework applicable to other drug-related adverse events, thereby contributing to the overall quality of clinical care.

## 2 Methods

### 2.1 Study design and reporting framework

This study is a narrative review that aims to provide a broad and thematically organized synthesis of the available evidence regarding nursing interventions for RAAEs. Although structured search strategies and established reporting frameworks such as SANRA were consulted to enhance rigor and transparency, this review does not constitute a systematic review or meta-analysis. Its primary objective is to synthesize existing knowledge, highlight clinical implications, and identify research gaps, rather than conduct a quantitative synthesis.

### 2.2 Literature search

To inform this narrative synthesis, a comprehensive search was undertaken in major databases, including PubMed, EMBASE, Scopus, Web of Science, CINAHL, and the China National Knowledge Infrastructure. The search strategy employed a combination of keywords such as “ribavirin,” “adverse events,” and “nursing intervention.” Eligible publications included articles in English and Chinese published up to March 1, 2025, with no date restrictions applied. The detailed search strategies are presented in Supplementary Table S1.

### 2.3 Eligibility considerations

Studies were deemed relevant if they investigated nursing interventions for RAAEs and adopted recognized clinical research designs such as randomized controlled trials, cohort studies, or retrospective analyses. Exclusion criteria encompassed case reports, qualitative studies, reviews, and meta-analyses lacking original data. These criteria served to guide the literature included in the narrative synthesis; however, no formal systematic selection or quantitative pooling was performed.

### 2.4 Evidence selection and synthesis

The literature search identified a substantial number of records. After removing duplicates and non-relevant studies, eligible publications focusing on nursing interventions for RAAEs were selected for in-depth review. The overall selection process is delineated in Supplementary Figure S1. It should be noted that this figure illustrates the comprehensiveness of the search rather than constituting a formal PRISMA-compliant process. Relevant studies were subsequently categorized and synthesized thematically, according to both the type of AEs (e.g., hematologic, gastrointestinal, neurological) and the intervention modality (e.g., preventive, symptomatic, supportive).

## 3 Overview of RAAEs

### 3.1 Pharmacological action and clinical indications of ribavirin

Ribavirin is a synthetic nucleoside analogue with broad-spectrum antiviral activity, primarily acting by inhibiting viral RNA-dependent RNA polymerase and disrupting viral replication (De Clercq, 2013). Clinically, it is used to treat chronic HCV infection, RSV infection, epidemic hemorrhagic fever, and certain viral hemorrhagic fevers (Snell, 2001). Although DAAs have largely replaced ribavirin in HCV treatment, the drug remains relevant in specific clinical scenarios, such as for patients with HCV genotype 3 or in resource-limited environments where DAAs may not be accessible (Pawlotsky, 2016). However, ribavirin’s clinical use is limited by its notable adverse effects, which pose significant challenges to its broader application.

### 3.2 Common adverse events and their clinical manifestations

Ribavirin is associated with a range of adverse events affecting multiple organ systems, often presenting with complex and varied clinical symptoms.

Hematologic toxicity: The most clinically significant adverse events of ribavirin is hemolytic anemia, which occurs in approximately 20%–30% of patients. This effect is mainly due to the accumulation of phosphorylated ribavirin metabolites in erythrocytes, leading to increased intracellular osmotic pressure, membrane fragility, and premature red blood cell destruction (De Franceschi et al., 2000). Ribavirin is also associated with leukopenia and thrombocytopenia, which may increase the risk of infection and bleeding (Fouad, 2013).

Gastrointestinal reactions: Nausea, vomiting, and diarrhea are common adverse events, affecting around 30%–40% of patients (Hadziyannis et al., 2004). In severe cases, these symptoms can cause dehydration and electrolyte imbalances, which may compromise patient health and treatment adherence (Hadziyannis et al., 2004).

Neurological symptoms: Headache, fatigue, and insomnia are frequently reported during ribavirin therapy (Hadziyannis et al., 2004; Sulkowski et al., 2004). These symptoms may result from direct effects on the central nervous system or from hypoxia secondary to anemia (Hadziyannis et al., 2004; Sulkowski et al., 2004).

Other adverse reactions: Cutaneous reactions, such as rash and pruritus, have been observed, possibly due to immune-mediated hypersensitivity (Hadziyannis et al., 2004). In addition, hepatic abnormalities, including transient elevations in liver transaminase levels, may also occur, requiring regular liver function monitoring to prevent severe hepatotoxicity (Hadziyannis et al., 2004).

### 3.3 Mechanisms underlying RAAEs

**FIGURE 1:**
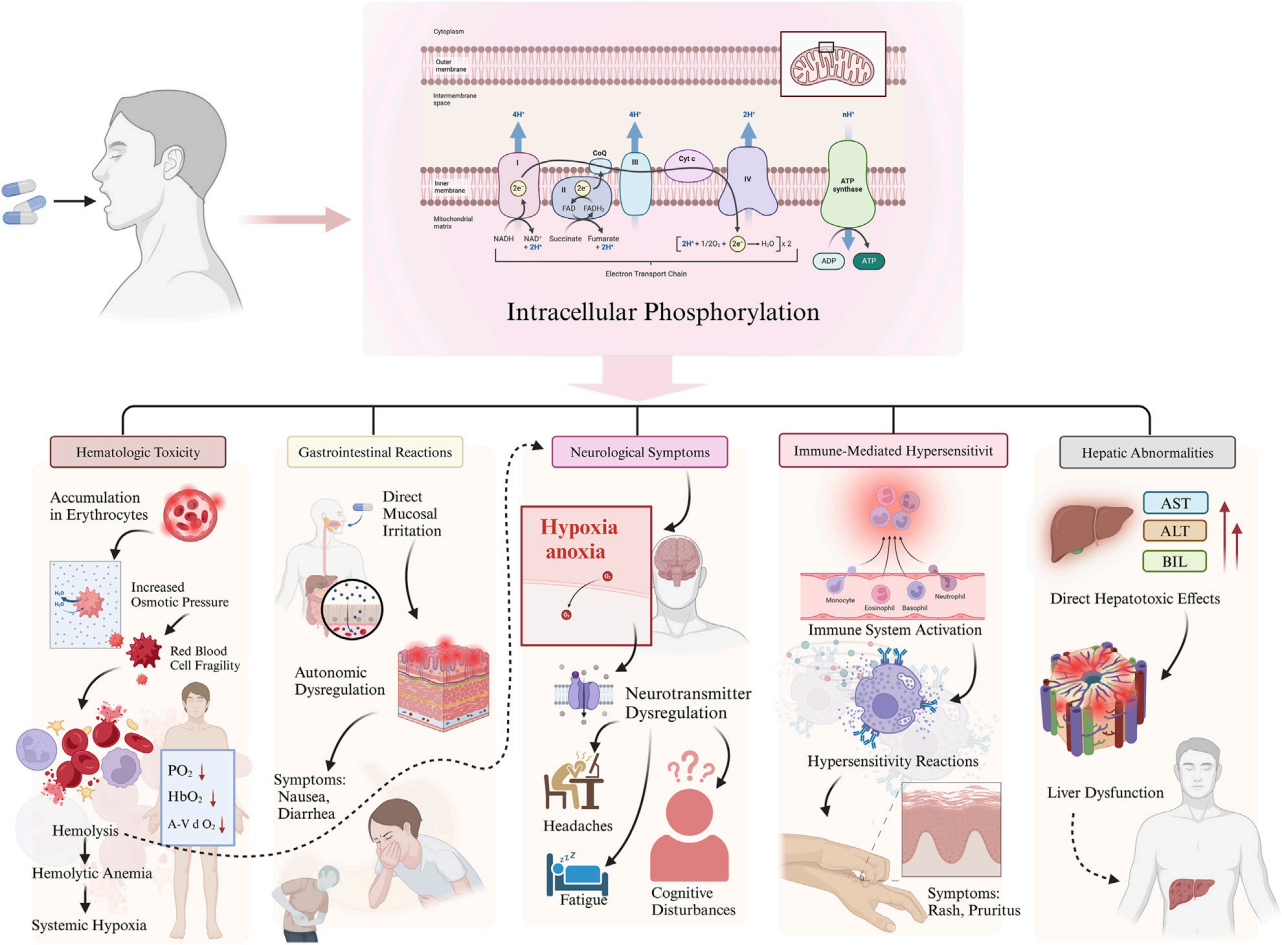
Mechanisms underlying ribavirin-associated adverse effects (RAAEs).

The pathophysiology of RAAEs is multifactorial, involving complex biochemical and physiological mechanisms (Figure 1).

Hematologic toxicity: Ribavirin undergoes intracellular phosphorylation within erythrocytes, leading to accumulation of metabolites that increase osmotic pressure and compromise membrane stability, ultimately resulting in hemolysis (Figure 1) (De Franceschi et al., 2000; Noureddin and Ghany, 2010). The resulting anemia may cause systemic hypoxia and related complications.

Gastrointestinal reactions: Ribavirin may cause direct irritation of the gastrointestinal mucosa, leading to symptoms such as nausea and diarrhea (Figure 1) (Russo and Fried, 2003). Additionally, its effects on the central nervous system may contribute to autonomic dysfunction, further exacerbating gastrointestinal symptoms (Russo and Fried, 2003).

Neurological symptoms: Hypoxia secondary to ribavirin-induced anemia may lead to fatigue, headaches, and cognitive disturbances (Figure 1) (Russmann et al., 2006). Moreover, ribavirin may also disrupt neurotransmitter regulation, contributing to neurological adverse events (Russmann et al., 2006).

Other reactions: Immune-mediated hypersensitivity is believed to underlie dermatologic manifestations such as rash and pruritus. Hepatic abnormalities may result from the direct hepatotoxicity of ribavirin metabolites (Figure 1) (Russo and Fried, 2003).

### 3.4 Impact on treatment adherence and clinical outcomes

The adverse effects associated with ribavirin have a substantial impact on treatment adherence and therapeutic outcomes (Russo and Fried, 2003). Studies indicate that approximately 15%–20% of patients discontinue ribavirin prematurely due to intolerable adverse events, which can reduce viral suppression, increase the risk of disease recurrence, and potentially lead to viral resistance (McHutchison et al., 2002). In addition, severe complications such as hemolytic anemia may worsen existing comorbidities, prolong hospital stays, and increase healthcare costs (Russmann et al., 2006). Therefore, effective management of RAAEs is crucial for improving patient outcomes (Russmann et al., 2006; Fellay et al., 2010). Early identification of adverse events, prompt therapeutic intervention, and individualized treatment strategies—including dose adjustments, supportive care, and adjunctive therapies—are essential to reduce these risks and enhance the overall efficacy of ribavirin-based regimens (Russmann et al., 2006; Fellay et al., 2010).

## 4 Role of nursing interventions in managing RAAEs

### 4.1 Theoretical foundations of nursing interventions

The application of nursing interventions in managing RAAEs is grounded in two core theoretical frameworks:

Evidence-based nursing practice: This approach combines current research evidence, clinical expertise, and patient values to formulate scientifically validated and effective nursing strategies (Titler and Hughes, 2008; Ferrell et al., 2018). For example, evidence-based protocols for anemia management have been shown to significantly reduce the incidence of ribavirin-induced hemolytic anemia (De Franceschi et al., 2000; Russo and Fried, 2003).

Individualized care principles: Tailored interventions are developed based on patient-specific characteristics such as age, gender, and underlying medical conditions (Russo and Fried, 2003; Fellay et al., 2010). For instance, women of reproductive age require special counseling on contraception due to ribavirin’s teratogenic risks (Russo and Fried, 2003; Fellay et al., 2010).

### 4.2 Key components of nursing interventions

Nursing interventions for RAAEs include the following essential components:

Early assessment and monitoring: Prior to initiating therapy, nurses perform a comprehensive baseline evaluation, including complete blood counts and liver and renal function tests (Hadziyannis et al., 2004; Russo and Fried, 2003; McHutchison et al., 2002). During treatment, ongoing monitoring of hemoglobin, white blood cell counts, and other indicators is critical for early detection of adverse events.

Symptom management and supportive care: Targeted strategies are employed to manage specific adverse events. For example, patients with anemia may receive iron supplements, vitamin B12, or erythropoietin, while those experiencing gastrointestinal symptoms may benefit from dietary counseling or antiemetic medications (Hadziyannis et al., 2004; Russo and Fried, 2003).

Patient education and psychological support: Structured education programs enhance patients’ understanding of potential adverse events and provide them with self-monitoring tools and coping techniques (McHutchison et al., 2002). Concurrently, psychological support helps reduce anxiety and promote treatment confidence.

Multidisciplinary collaboration and comprehensive management: Nurses collaborate with physicians, pharmacists, dietitians, and other healthcare professionals to develop integrated care plans (Sulkowski et al., 2004; McHutchison et al., 2002). For example, pharmacists may assist with dose adjustments, and dietitians may contribute to individualized nutrition planning.

### 4.3 Objectives of nursing interventions

The goals of nursing interventions in managing RAAEs are multifaceted:

Reducing the severity of adverse events: Timely and effective nursing management can significantly lower the incidence and intensity of adverse events (Russo and Fried, 2003). For instance, regular monitoring and interventions have been shown to reduce severe anemia by 30%–50%.

Improving treatment adherence: Through education, support, and symptom control, nurses enhance patients’ engagement and commitment to therapy (McHutchison et al., 2002). Evidence suggests that systematic nursing interventions can improve treatment adherence by more than 20% (McHutchison et al., 2002).

Enhancing patient quality of life: By alleviating symptoms, offering emotional support, and optimizing care plans, nursing interventions significantly improve both physical and psychological well-being (Hadziyannis et al., 2004; Sulkowski et al., 2004). Interventions targeting fatigue and insomnia, for example, have been shown to enhance daily functioning and sleep quality.

## 5 Clinical research on nursing interventions for RAAEs

### 5.1 Current status of international and domestic research

#### 5.1.1 International research progress and achievements

International studies have made significant advances in managing adverse reactions associated with ribavirin and interferon combination therapy for chronic hepatitis C (CHC). Research confirms that pegylated interferon α-2a (PEG-IFNα-2a) combined with ribavirin remains a standard treatment for CHC, but it is associated with a high incidence of adverse events, including flu-like symptoms, hematologic toxicity (e.g., leukopenia and thrombocytopenia), gastrointestinal symptoms (e.g., nausea, vomiting), and neurological disturbances (e.g., depression, anxiety). International research emphasizes multidisciplinary collaboration and individualized nursing interventions to reduce adverse event incidence, improve treatment adherence, and enhance patient quality of life. For example, guidelines from the American Association for the Study of Liver Diseases and the European Association for the Study of the Liver explicitly recommend nursing interventions for managing adverse events, including psychological support, symptom management, and patient education.

The classification of nursing interventions in this review follows a clinically validated framework, aligning with the World Health Organization’s Adverse Event Management Guidelines (2023) and the Nursing Intervention Taxonomy (NIT-2.0). Findings are systematically grouped by (a) adverse event type–hematologic, gastrointestinal, and neurological–and (b) intervention modality–preventive, symptomatic, and rehabilitative. This dual-axis categorization ensures methodological consistency with established clinical protocols while facilitating direct translation of research findings into clinical practice.

#### 5.1.2 Domestic research status and limitations

Research on nursing interventions for RAAEs in China began relatively late but has received growing attention in recent years (Fan et al., 2016; Feng, 2015). Multiple studies report that domestic nursing practice mainly focuses on symptom control and psychological support (Jia and He, 2021; Sun, 2021; Liu and Wu, 2017; Zhang and Chang, 2015; Zhang and Shen, 2013; He, 2012; Zhang et al., 2014; Hui, 2010; Tan, 2011) (Table 1). For instance, Zhang and Chang (2015) conducted an observational study involving 74 CHC patients and found that comprehensive nursing interventions significantly reduced the incidence of adverse events and improved treatment adherence. Similarly, in a single-center study, Sun (2021) also observed improved patient adherence following the implementation of comprehensive nursing interventions. Although these results are encouraging, it should be noted that they are derived from observational studies conducted at a single institution with limited sample sizes. Consequently, the generalizability of the findings remains constrained, highlighting the necessity for further validation through larger, multi-center RCTs.

**TABLE 1:** Summary of eligible studies.

Reference	Sample size	Type of study	Participants	Treatment modalities	Findings	Methodological limitations
Jia and He (2021)	80	Randomized controlled trial	Patients with CHCV	Pegylated interferon + ribavirin + nursing intervention	Nursing intervention showed higher virological response rates, lower ALT/AST levels, fewer adverse reactions, and higher satisfaction	Single-center study, relatively small sample size, potential lack of blinding
Sun (2021)	90	Observational study	Patients with CHCV	Pegylated interferon + ribavirin + nursing intervention	Nursing intervention improved treatment compliance and reduced negative emotions	Single-center, non-randomized design, potential selection bias, limited sample size
Liu and Wu (2017)	106	Observational study	Patients with CHCV	Pegylated interferon + ribavirin + nursing intervention	Nursing intervention improved psychological state post-treatment	Single-center, non-randomized design, potential selection bias
Zhang and Chang (2015)	74	Observational study	Patients with CHCV	Pegylated interferon + ribavirin + nursing intervention	Nursing intervention improved compliance and treatment completion	Single-center, non-randomized design, small sample size, lack of a control group
Zhang and Shen (2013)	60	Observational study	Patients with CHCV	Pegylated interferon + ribavirin + nursing intervention	Nursing intervention improved compliance and satisfaction	Single-center, non-randomized design, small sample size
He (2012)	35	Retrospective study	Patients with CHCV	Pegylated interferon + ribavirin + nursing intervention	Nursing intervention enhanced patients’ compliance and achieve better therapeutic outcomes	Single-center, very small sample size, retrospective design carries risks of recall bias and incomplete data
Zhang et al. (2014)	132	Observational study	Patients with CHCV	Pegylated interferon + ribavirin + nursing intervention	Nursing intervention improved adherence to treatment	Single-center, non-randomized design
Hui (2010)	39	Observational study	Patients with CHCV	Pegylated interferon + ribavirin + nursing intervention	Nursing intervention enhances medication adherence, ensuring treatment completion	Single-center, non-randomized design, small sample size
Tan (2011)	42	Observational study	Patients with CHCV	Pegylated interferon + ribavirin + nursing intervention	Nursing intervention ensured successful treatment completion and excellent outcomes	Single-center, non-randomized design, small sample size

TG, treatment group; CG, control group; CHCV, chronic hepatitis C virus; ALT, Alanine Aminotransferase; AST, Aspartate Aminotransferase.

Beyond the constraints in study design and scale, several other limitations persist in the domestic research landscape. First, there is a notable scarcity of large-sample, multi-center RCTs, which limits the robustness of evidence. Second, the systematic and standardized application of nursing interventions requires further development, with some studies lacking a rigorous evidence-based framework. Third, research focusing on special populations—such as the elderly and pediatric patients—remains insufficient and merits expanded investigation in future studies.

### 5.2 Specific measures and effectiveness evaluation of nursing interventions

#### 5.2.1 Nursing interventions for hematologic toxicity

Hematologic toxicity is a common adverse event associated with ribavirin and interferon therapy, primarily manifesting as leukopenia, thrombocytopenia, and anemia. Nursing interventions include routine monitoring of blood cell counts, timely dose adjustments, and supplementation with hematopoietic agents such as iron and vitamin B12 (Supplementary Table S2). Studies have demonstrated that early, individualized nursing interventions can significantly reduce hematologic toxicity and minimize treatment discontinuation. For example, Jia and He (2021) reported that patients receiving combination therapy with ribavirin and PEG-IFNα-2a experienced significantly lower rates of bone marrow suppression and other hematologic adverse events compared to the control group.

#### 5.2.2 Nursing interventions for gastrointestinal reactions

Gastrointestinal reactions, such as nausea, vomiting, and loss of appetite, are common adverse events of ribavirin therapy. Nursing interventions include dietary guidance (e.g., small, frequent meals and avoidance of greasy foods), medication adjustments (e.g., administration of antiemetics), and psychological support (Supplementary Table S2). Liu and Wu (2017) demonstrated that comprehensive nursing interventions significantly alleviated gastrointestinal symptoms and improved treatment adherence. In addition, Zhang and Shen (2013) reported that gastrointestinal reactions are frequent adverse events of peginterferon-ribavirin therapy, and nursing interventions effectively reduced symptom severity.

#### 5.2.3 Nursing interventions for neurological symptoms

Neurological symptoms, including depression, anxiety, and insomnia, are serious adverse events of ribavirin combined with interferon therapy. Nursing interventions encompass psychological counseling, pharmacological support (e.g., antidepressants), and lifestyle modifications such as maintaining regular sleep patterns and engaging in moderate physical activity (Supplementary Table S2). Zhang and Shen (2013) found that psychological nursing and health education significantly improved neurological symptoms and promoted treatment adherence. He (2012) reported a 20% incidence of neuropsychiatric symptoms, which could be effectively reduced through targeted nursing interventions.

#### 5.2.4 Application of comprehensive nursing interventions

Comprehensive nursing interventions involve multidisciplinary collaboration and integrate symptom management, psychological support, and health education to improve both treatment adherence and quality of life (Supplementary Table S2). Multiple studies have shown that comprehensive nursing approaches significantly reduce the incidence of adverse events and enhance clinical outcomes. For example, Jia and He (2021) found that comprehensive nursing interventions improved patient satisfaction and reduced the frequency of adverse events. Similarly, Sun (2021) highlighted that such interventions effectively alleviated negative emotions and improved treatment adherence in patients undergoing antiviral therapy.

This study adopts a mechanism-to-intervention mapping strategy. For each major RAAE category (e.g., ribavirin-induced hemolysis), interventions are presented in a hierarchical sequence: (1) pathophysiological basis, (2) evidence-based nursing actions, and (3) outcome metrics. This structure reflects the clinical decision-making process, aligning intervention selection with underlying mechanisms and expected therapeutic endpoints.

A critical synthesis of the available evidence, as detailed in Supplementary Table S2, indicates that while diverse nursing interventions show potential in managing RAAEs, the current findings are primarily based on small-scale, single-center studies conducted in China. To facilitate a transparent evaluation of the evidence supporting each intervention, the study design, sample size, main findings and methodological constraints are summarized in Table 1. This summary enables readers to critically assess the robustness and generalizability of the reported benefits.

### 5.3 Clinical research designs and methods for nursing interventions

RCTs are considered the gold standard for evaluating the effectiveness of nursing interventions. Randomization and the use of control groups help minimize bias and enhance the credibility of findings. For example, Sun (2021) used an RCT to evaluate the impact of comprehensive nursing interventions on treatment adherence in CHC patients, demonstrating significantly higher adherence in the intervention group. Similarly, Jia and He (2021) employed an RCT design and found notable improvements in virological response rates and patient satisfaction among patients receiving nursing interventions.

Cohort studies are appropriate for assessing the long-term effects of nursing interventions. Prospective designs allow researchers to evaluate intervention impact on clinical outcomes over time. For instance, Zhang et al. (2014) conducted a cohort study to examine the effect of nursing interventions on adverse events in CHC patients, reporting a significant reduction in adverse event rates. Zhang and Chang (2015) also used a cohort design and found that comprehensive nursing interventions markedly improved treatment adherence.

Case-control studies are useful for examining associations between nursing interventions and specific clinical outcomes. Retrospective designs enable more rapid data collection and analysis. For example, Hui (2010) used a case-control design to explore the relationship between nursing interventions and treatment adherence in CHC patients, showing substantially higher adherence in the intervention group.

It is essential to interpret the findings of the aforementioned studies in light of their inherent methodological limitations. While RCTs represent the highest standard of evidence, it is noteworthy that the majority of included domestic investigations—such as those conducted by Zhang and Chang (2015) and Sun (2021)—adopt observational designs (e.g., cohort or case-control studies). These are inherently more susceptible to biases than RCTs. Therefore, although the positive outcomes reported are promising, they should be regarded as preliminary and warrant further validation through larger, rigorously conducted multicenter RCTs.

### 5.4 Evidence-based support and practice guidelines for nursing interventions

Currently, multiple international and domestic guidelines support the implementation of nursing interventions for RAAEs. For instance, the AASLD and EASL guidelines explicitly recommend nursing strategies such as symptom management, psychological support, and health education. In China, the *Guidelines for the Prevention and Treatment of CHC* also provided practical recommendations for nursing care. Zhang and Chang (2015) noted that interferon-ribavirin therapy frequently induces flu-like symptoms and bone marrow suppression, and that comprehensive nursing interventions significantly improved patient adherence. Tan LJ also emphasized the role of nursing interventions in reducing the incidence of adverse events (Tan, 2011).

In summary, considerable progress has been made in the research and practice of nursing interventions for RAAEs both internationally and domestically. However, limitations remain. Future studies should prioritize high-quality research designs; standardized and systematic nursing protocols, and targeted approaches for special populations to further enhance treatment outcomes and quality of life for patients with CHC.

### 5.5 Limitations and research gaps

Despite the growing body of research on nursing interventions for RAAEs, several critical limitations persist. First, many studies suffer from methodological weaknesses, such as small sample sizes, lack of randomization, absence of blinding, and insufficient use of control groups. These limitations undermine internal validity and reduce the reliability of outcome assessments. Second, there is considerable heterogeneity in intervention protocols, outcome indicators, and evaluation criteria, which complicates cross-study comparisons and hinders the development of standardized clinical guidelines. Third, only a limited number of studies have included long-term follow-up, making it difficult to assess the sustained impact of nursing interventions on patient-centered outcomes such as quality of life, recurrence of adverse events, and healthcare utilization. Additionally, this narrative review focused exclusively on quantitative evidence, leaving unexplored the nuanced perspectives of nurses and patients regarding intervention acceptability, clinical experiences, and contextual implementation challenges—key factors influencing real-world effectiveness.

While the proposed taxonomy of interventions provides operational clarity, it inevitably simplifies complex clinical realities. For example, fatigue—classified under neurological symptoms—may simultaneously originate from anemia (hematologic) and depression (psychological). Such overlaps underscore the need for analytical approaches capable of capturing multi-domain interdependencies. Future research should consider employing network analysis methods to quantify these cross-domain interactions, thereby enabling a more integrated understanding of RAAEs pathophysiology and nursing management.

To address these gaps, future studies should prioritize high-quality, multicenter, and longitudinal designs. Efforts should focus on developing standardized protocols, tailoring interventions for diverse populations (including elderly and pediatric patients), and incorporating economic evaluations to ensure feasibility and sustainability. A mixed-methods approach is particularly warranted to: (1) explore nurses’ clinical decision-making processes in managing RAAEs, (2) evaluate patient-reported experiences and intervention acceptability, and (3) identify contextual barriers and facilitators to implementation. Such qualitative insights would complement efficacy data and strengthen translational potential. These efforts are essential for translating evidence into practice and enhancing the consistency, accessibility, and quality of care for patients receiving ribavirin therapy.

## 6 Comparison with previous studies

This narrative review emphasizes the critical role of nursing interventions in mitigating RAAEs. The evidence shows that structured, evidence-based strategies—including early monitoring, individualized symptom management, and multidisciplinary collaboration—significantly improve treatment adherence and patient quality of life. These observations align with findings from international studies and established guidelines. For instance, Larrey et al. reported that nurse-led education enhanced adherence and reduced the need for dose modifications (Larrey et al., 2011), whereas Cacoub et al. demonstrated that education programs increased treatment persistence (Cacoub et al., 2008). Similarly, AASLD and EASL guidelines underscore the importance of incorporating nursing interventions into the management of ribavirin-induced adverse events.

By contrast, domestic studies have reported positive outcomes, but their evidence is limited by small sample sizes and methodological weaknesses. For example, Zhang and Chang (2015) and Sun (2021) found that comprehensive nursing care reduced gastrointestinal and neurological symptoms, thereby enhancing adherence. However, unlike international multicenter trials, most domestic research is observational and lacks rigorous randomization or blinding, which weakens the strength of the evidence.

Moreover, although both domestic and international research support the effectiveness of nursing interventions, international studies more frequently adopt multidisciplinary approaches—such as collaboration with pharmacists and dietitians—to address the complex nature of RAAEs. This indicates a gap in practice and research that should be addressed in future Chinese studies.

Overall, the results of this review are consistent with existing literature, reinforcing the essential role of nursing interventions in ensuring the safe and effective management of ribavirin therapy. Nevertheless, discrepancies between domestic and international research highlight the need for large-scale multicenter trials and standardized nursing protocols in China. Bridging these gaps will allow future studies to strengthen global consensus and enhance the quality of patient care in diverse healthcare settings.

## 7 Challenges and future directions for nursing interventions

### 7.1 Current challenges in nursing interventions

Nursing interventions for RAAEs face several significant challenges that limit their effectiveness and broader clinical adoption. First, the complexity of individual patient characteristics- including age, gender, comorbidities, and genetic predispositions- complicates the development of personalized care strategies (Larrey et al., 2011). For example, elderly patients and women of reproductive age often demonstrate reduced tolerance to adverse events, requiring highly individualized interventions. Second, limited nursing resources, such as workforce shortages and inadequate access to specialized equipment, present major barriers, especially in primary healthcare settings where nurses may lack training specific to RAAEs management (Russmann et al., 2006; De Clercq, 2013). Third, there is a lack of high-quality, large-scale RCTs to establish robust evidence-based support (Russmann et al., 2006). Most existing studies are focused on single symptoms, with insufficient long-term follow-up, hindering the development of comprehensive and standardized nursing guidelines. These challenges highlight the urgent need for innovative and further research to optimize nursing interventions in this area.

### 7.2 Future research directions

To overcome these challenges and advance the field, future research should prioritize the following areas:

Developing standardized nursing interventions protocols: Establishing evidence-based, structured protocols for managing RAAEs is essential. These protocols should include early assessment, symptom management, patient education, and multidisciplinary collaboration to promote consistency and practical implementation in clinical settings.

Conducting multicenter, large-sample clinical studies: High-quality, multicenter trials with sufficient sample sizes are crucial to validate the effectiveness and generalizability of nursing interventions. Long-term follow-up should be included to assess outcomes such as treatment adherence and patient quality of life.

Exploring innovative nursing models: The integration of information technology and artificial intelligence offers opportunities to transform nursing practice. For instance, remote nursing platforms and intelligent monitoring tools, such as wearable devices for real-time hemoglobin tracking, may support timely care adjustments and improved outcomes.

Enhancing professional training and capacity building: Expanding specialized education programs for nurses is vital to enhance their competencies in managing RAAEs. Promoting nurse involvement in research and fostering a culture of continuous professional development will drive innovation and elevate the quality of care.

## 8 Summary

In recent years, significant progress has been made in nursing interventions for the management of RAAEs. Research shows that for hematologic toxicity, particularly hemolytic anemia, routine hemoglobin monitoring, appropriate dose adjustments, and nutritional support can effectively reduce incidence and improve patient tolerance. Gastrointestinal adverse events, such as nausea, vomiting, and diarrhea, can be managed through evidence-based nursing strategies, including dietary counseling, pharmacological interventions, and psychological support, all of which help enhance treatment adherence. In addition, neurological symptoms-including fatigue, headaches, and insomnia- can be alleviated through lifestyle modifications, anxiety management, and individualized nursing care, thereby improving patients’ overall quality of life. Given the substantial impact of adverse effects on treatment adherence and clinical outcomes, comprehensive nursing interventions are essential for optimizing therapeutic efficacy. Future research should focus on precision-based and multidisciplinary approaches, integrating predictive biomarkers and digital health technologies, such as remote monitoring and artificial intelligence-assisted decision-making, to further improve intervention effectiveness. Large-scale, multicenter clinical trials are needed to validate the long-term benefits and safety of these nursing strategies and promote a more standardized, patient-centered approach in clinical practice.

## Data Availability

The original contributions presented in the study are included in the article/Supplementary Material, further inquiries can be directed to the corresponding author.

## Abbreviations

HCV, hepatitis C virus; RSV, respiratory syncytial virus; DAAs, direct-acting antivirals; RAAEs, Ribavirin-Associated Adverse Events; CNKI, China National Knowledge Infrastructure; RCTs, randomized controlled trials; CHC, chronic hepatitis C; PEG-IFNα-2a, pegylated interferon α-2a.
