# Supplementary material for: Advances in clinical application on nursing intervention for ribavirin-associated adverse events
Source: Front Pharmacol. 2025 Oct 28;16:1596007. doi: 10.3389/fphar.2025.1596007 (PMC12602387; doi:10.3389/fphar.2025.1596007)

Supplementary Table S1. Detailed search strategies

**PubMed**	**#1**: "ribavirin" [MeSH Terms] OR "ribavirin"[Title/Abstract]<br>**#2**: "adverse effects"[Title/Abstract] OR "adverse events"[Title/Abstract] OR "side effects"[Title/Abstract]<br>**#3**:"nursing"[MeSH Terms] OR "nursing care"[Title/Abstract] OR "nursing intervention" [Title/Abstract] OR "nursing practice" [Title/Abstract]<br>**#4**:"randomized controlled trials as topic"[MeSH Terms] OR "randomized controlled trial" [Publication Type] OR "cohort studies" [MeSH Terms] OR "case-control studies"[MeSH Terms] OR "retrospective studies" [MeSH Terms] OR "clinical trial" [Publication Type]<br>**#5**: (#1 AND #2 AND #3) AND #4
**EMBASE**	**#1**:"ribavirin"/exp OR "ribavirin": ab,ti<br>**#2**:"adverse effects": ab,ti OR "adverse events": ab,ti OR "side effects": ab,ti<br>**#3**:"nursing"/exp OR "nursing care"/exp OR "nursing intervention": ab,ti OR "nursing practice":ab,ti<br>**#4**:"random allocation"/exp OR "randomized controlled trial"/exp OR "allocation, random"/exp OR "randomization"/exp<br>**#5**: (#1 AND #2 AND #3) AND #4
**Web of Science**	**#1:**TS= (ribavirin)<br>**#2:**TS= (adverse effects OR adverse events OR side effects)<br>**#3:**TS= (nursing OR nursing care OR nursing intervention OR nursing practice)<br>**#4:**TS= (random allocation OR randomized controlled trial OR allocation, random OR randomization)<br>**#5:** (#1 AND #2 AND #3) AND #4
**Scopus**	**#1:** TITLE-ABS-KEY (ribavirin) OR KEY (ribavirin)<br>**#2:** KEY (adverse effects OR adverse events OR side effects)<br>**#3:** TITLE-ABS-KEY (nursing OR nursing care) OR KEY (nursing intervention OR nursing practice)<br>**#4**: TITLE-ABS-KEY (random allocation OR randomized controlled trial

	OR allocation, random OR randomization)<br>**#5**: (#1 AND #2 AND #3) AND #4
**CINAHL**	**#1**:AB (ribavirin) AND TX (ribavirin)<br>**#2**:TX (adverse effects OR adverse events OR side effects)<br>**#3**:AB (nursing OR nursing care) AND TX (nursing intervention OR nursing practice)<br>**#4**: AB (random allocation OR randomized controlled trial OR allocation, random OR randomization)<br>**#5**: (#1 AND #2 AND #3) AND #4
**CNKI**	**#1**: TKA%=“利巴韦林”<br>**#2**: TKA%=“不良反应” OR TKA%=“不良事件” OR TKA%=“副作用”<br>**#3**: TKA%=“护理干预” OR TKA%=“护理措施” OR TKA%=“护理研究”<br>**#4**: TKA%=“随机对照试验” OR TKA%=“临床试验” OR TKA%=“前瞻性研究” OR TKA%=“回顾性研究”<br>**#5**: (#1 AND #2 AND #3) AND #4

Notes: China National Knowledge Infrastructure.

Supplementary Table S2. Summary of nursing interventions for RAAEs

**Adverse event category**	**Pathophysiological basis**	**Key nursing interventions**	**Reported outcomes**
Hematologic toxicity (e.g., hemolytic anemia, leukopenia,thrombocytopenia)	Ribavirin metabolite accumulation in erythrocytes → osmotic stress & hemolysis	Routine CBC monitoring; dose adjustment; iron, vitamin B12, or erythropoietin supplementation	Reduced incidence of severe anemia by 30 – 50%; improved treatment continuation
Gastrointestinal reactions (nausea, vomiting, diarrhea, anorexia)	Direct mucosal irritation; central nervous system/autonomic dysfunction	Dietary counseling (small, frequent meals; avoid greasy foods); antiemetic use; psychological support	Improved adherence; reduced symptom severity
Neurological symptoms (fatigue, insomnia, depression, anxiety)	Hypoxia secondary to anemia; neurotransmitter dysregulation	Psychological counseling; antidepressant therapy if needed; lifestyle modification (sleep hygiene, physical activity)	Alleviation of neuropsychiatric symptoms; better quality of life; improved adherence
Other reactions (rash, pruritus, hepatic dysfunction)	Immune-mediated hypersensitivity; hepatotoxic metabolites	Skin care guidance; antihistamines; regular liver function monitoring	Reduced dermatologic discomfort; early detection of hepatotoxicity; enhanced patient safety
Comprehensive interventions	Multisystem toxicity; overlapping pathophysiological pathways	Multidisciplinary collaboration (physicians, pharmacists, dietitians); patient education; continuous follow-up	Improved patient satisfaction; lower discontinuation rates; enhanced treatment efficacy

Notes: RAAEs,Ribavirin-Associated Adverse Events; CBC, Complete Blood Count.

Supplementary Figure S1. Flow diagram of study selection

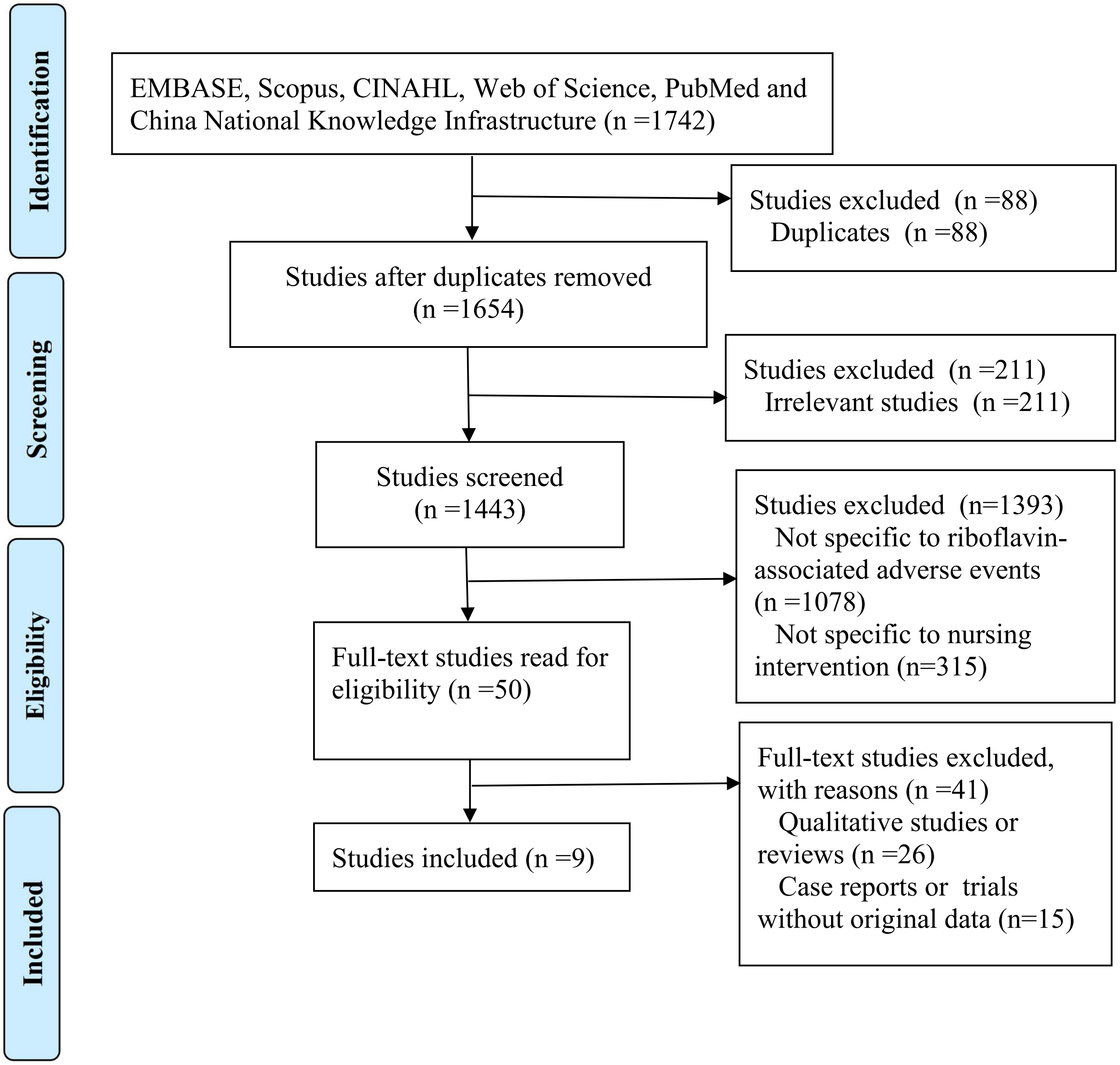

Supplement: Supplementary file 1 [file DataSheet1.pdf]
